# A glucotolerant β-glucosidase from the fungus *Talaromyces amestolkiae* and its conversion into a glycosynthase for glycosylation of phenolic compounds

**DOI:** 10.1186/s12934-020-01386-1

**Published:** 2020-06-10

**Authors:** Juan Antonio Méndez-Líter, Manuel Nieto-Domínguez, Beatriz Fernández de Toro, Andrés González Santana, Alicia Prieto, Juan Luis Asensio, Francisco Javier Cañada, Laura Isabel de Eugenio, María Jesús Martínez

**Affiliations:** 1 Department of Microbial and Plant Biotechnology, Centro de Investigaciones Biológicas, CSIC, Ramiro de Maeztu 9, 28040 Madrid, Spain; 2 Department of Chemical and Physical Biology, Centro de Investigaciones Biológicas, CSIC, Ramiro de Maeztu 9, 28040 Madrid, Spain; 3 Glycochemistry and Molecular Recognition Group, Instituto de Química Orgánica General (IQOG-CSIC), Calle Juan de la Cierva, 3, 28006 Madrid, Spain

**Keywords:** Transglycosylation, Glycosynthases, Glycoside hydrolases, Phenolic compounds

## Abstract

**Background:**

The interest for finding novel β-glucosidases that can improve the yields to produce second-generation (2G) biofuels is still very high. One of the most desired features for these enzymes is glucose tolerance, which enables their optimal activity under high-glucose concentrations. Besides, there is an additional focus of attention on finding novel enzymatic alternatives for glycoside synthesis, for which a mutated version of glycosidases, named glycosynthases, has gained much interest in recent years.

**Results:**

In this work, a glucotolerant β-glucosidase (BGL-1) from the ascomycete fungus *Talaromyces amestolkiae* has been heterologously expressed in *Pichia pastoris*, purified, and characterized. The enzyme showed good efficiency on *p*-nitrophenyl glucopyranoside (*p*NPG) (*K*_*m*_= 3.36 ± 0.7 mM, *k*_*cat*_= 898.31 s^−1^), but its activity on cellooligosaccharides, the natural substrates of these enzymes, was much lower, which could limit its exploitation in lignocellulose degradation applications. Interestingly, when examining the substrate specificity of BGL-1, it showed to be more active on sophorose, the β-1,2 disaccharide of glucose, than on cellobiose. Besides, the transglycosylation profile of BGL-1 was examined, and, for expanding its synthetic capacities, it was converted into a glycosynthase. The mutant enzyme, named BGL-1-E521G, was able to use α-d-glucosyl-fluoride as donor in glycosylation reactions, and synthesized glucosylated derivatives of different *p*NP-sugars in a regioselective manner, as well as of some phenolic compounds of industrial interest, such as epigallocatechin gallate (EGCG).

**Conclusions:**

In this work, we report the characterization of a novel glucotolerant 1,2-β-glucosidase, which also has a considerable activity on 1,4-β-glucosyl bonds, that has been cloned in *P. pastoris*, produced, purified and characterized. In addition, the enzyme was converted into an efficient glycosynthase, able to transfer glucose molecules to a diversity of acceptors for obtaining compounds of interest. The remarkable capacities of BGL-1 and its glycosynthase mutant, both in hydrolysis and synthesis, suggest that it could be an interesting tool for biotechnological applications.

## Introduction

Lignocellulosic biomass is the most abundant renewable carbon source in the world, and it is useful as raw material to produce value-added products for the benefit of mankind. It is composed for three distinct polymers: lignin, cellulose, and hemicelluloses. Cellulose is the major polysaccharide in plant cell-wall and it is a linear homopolymer of β-1,4-glucose. For a long time, the degradation of this polysaccharide was exclusively attributed to the synergistic action of cellulolytic glycoside hydrolases (GHs): endoglucanases (EGs), cellobiohydrolases (CBHs), and β-glucosidases (BGLs) [1]. However, this classic model underwent a big breakthrough with the discovery of lytic polysaccharide monooxygenases (LPMOs), auxiliary enzymes that break crystalline cellulose by redox reactions, thus creating new chains, and improving cellulose accessibility by glycoside hydrolases [2]. The combination of endoglucanases, cellobiohydrolases and LPMOs generates cellobiose and other small cellooligosaccharides, which are finally degraded to glucose by β-glucosidases. Hence, BGLs are fundamental to complete the breakage of this polysaccharide up to the monosaccharide level since, if they are not in sufficient amount, short chain oligosaccharides accumulate, inhibiting the other cellulases and decreasing total yields [3]. However, BGLs are generally found in low proportion in commercial preparations (mostly obtained from *Trichoderma reesei*) which usually represents the major bottleneck in cellulose degradation. Therefore, many studies are focused on finding robust and efficient β-glucosidases in order to supplement cellulolytic cocktails with this activity to increase the efficiency of cellulose saccharification. BGLs are widely distributed in GHs families, but, according to the CAZy database, they are mostly located in the GH1 and GH3 families, being GH1 the one with the largest number of characterized BGLs [4].

These enzymes are frequently inhibited by their own product, glucose, and finding glucotolerant BGLs could be of special interest. This would allow reducing the enzyme amount required for complete saccharification, and consequently, process costs. Despite their high hydrolytic capacity, glucotolerant GH3 BGLs are exceptional [5] but, on the contrary, this desirable property is common among GH1 BGLs.

On the other side, the capacity of GHs for catalyzing transglycosylation reactions makes them a great biotechnological tool for the synthesis or modification of molecules of interest, through the addition of one or more sugar units to different compounds. Increasing the solubility of the original compound, making it safer, or improving its stability are among the many beneficial effects reported for glycosides [6, 7]. The application of biocatalysis to produce new glycosides has two main advantages compared to classical chemical synthesis: (i) the process is generally much less polluting and more ecological, and (ii) it is usually simpler, since the complex structure of oligosaccharides makes the chemical approach difficult as the stereospecificity and regiospecificity of the glycosylation need to be controlled [8].

Although GHs can be successfully used to synthesize glycoconjugates, the yields are often poor. The reaction proceeds under kinetic control and the newly formed products are easily hydrolyzed by the same GH when the initial glycoside donor is exhausted making the process economically unfeasible at a large-scale. However, the hydrolytic capacity of these enzymes can be eliminated using rational design to produce novel biocatalysts with only synthetic activity. In this context, directed mutagenesis of one of the two catalytic residues placed in the active center of GHs, the nucleophile amino acid, abolishes their hydrolytic activity [9]. The resulting mutants, called glycosynthases, catalyze the formation of glycosidic bonds, using glycosyl fluoride donors as surrogates of the enzyme covalent intermediate and as they cannot degrade the newly-synthetized glycosides, the reaction yields are much higher [10]. The use of glycosynthases has been shown as an effective way to generate a wide variety of value-added products, as oligosaccharides of nitrophenol-sugars, methylumbellyferyl-sugars [8], oligosaccharides [11], and human milk oligosaccharides with prebiotic activity [12].

The ascomycete *T. amestolkiae* was studied for its ability to degrade cellulose and hemicellulose. Sequencing and annotation of the genome of this fungus disclosed a high number of genes encoding GHs and specially BGLs, having a number of CAZymes significantly higher than reported for other organisms used to produce commercial cellulolytic enzyme cocktails [13]. Two of the BGLs produced by *T. amestolkiae*, BGL-2 and BGL-3, belong to the GH3 family and have interesting peculiarities. BGL-2 is the major β-glucosidase secreted by this fungus in the presence of cellulosic inducers. Structurally, this enzyme has a cellulose binding domain, an unusual feature among BGLs [14]. On the other hand, *T. amestolkiae* produces BGL-3 in all carbon sources tested, which is uncommon for β-glucosidases, and this catalyst was isolated and characterized from a basal medium with glucose under carbon starvation conditions [15]. Both, BGL-2 and BGL-3, were used as BGL supplements of commercial cocktails for saccharification of lignocellulosic waste, but its inhibition by product is a handicap to improve the performance of the process. Up to now, the BGLs from the family GH1 produced by this fungus remain completely unexplored. In this work, we report the cloning and expression in *Pichia pastoris* of the β-glucosidase gene *bgl1* of this fungus, the purification and characterization of the recombinant BGL-1 and its classification as a GH1 BGL, as well as its conversion into a glycosynthase to expand the synthetic applications of this enzyme.

## Results

### Cloning, production, purification and biochemical characterization of BGL-1

In a previous work [13], the secretome released by *T. amestolkiae* growing in different carbon sources was analyzed and, in every condition tested, one potential BGL from GH1 family (protein g8384, renamed as BGL-1) was detected in very low amounts. After identifying the DNA sequence, RNA was extracted from 7-day-old cultures of *T. amestolkiae* growing in Mandels medium induced with Avicel as carbon source, obtaining total cDNA by retrotranscription. The sequence of the mature *bgl*-*1* comprised 1906 bp, including one intron, and encodes a 619 amino acids protein. Therefore, the *bgl1* gene, without the intron, was cloned and expressed in *P. pastoris* to increase BGL-1 production for analyzing its glucose tolerance, kinetic and physicochemical properties. The transformants were screened to detect the best β-glucosidase producers. The maximal β-glucosidase activity reached was 75 U/mL in 9-day-old cultures in YEPS liquid medium. The protein was purified from crude extracts of *P. pastoris*, at laboratory scale, in very high yields (recovered activity: 80% of BGL, purification degree: 5.89) after just one step of anion-exchange chromatography.

The isoelectric point of the pure BGL-1 was 6.7 and its molecular mass, determined by MALDI-TOF mass spectrometry, was 88.11 kDa, 23% higher (68.05 kDa) than expected from its amino acid sequence. The differences can be attributed to the hyperglycosylation of the *P. pastoris* proteins [16]. Maximum activity of BGL-1 in 10 min reactions was found at pH 4 and 60 °C, similar values to those reported for other native β-glucosidases [3, 17], but BGL-1 works unusually well at more acidic pHs. This behavior was also observed in the BGL-2 and BGL-3 characterized from this fungus, both from GH3 family [14, 15].

### Glucose tolerance, kinetic study and substrate specificity of BGL-1

**Fig. 1:**
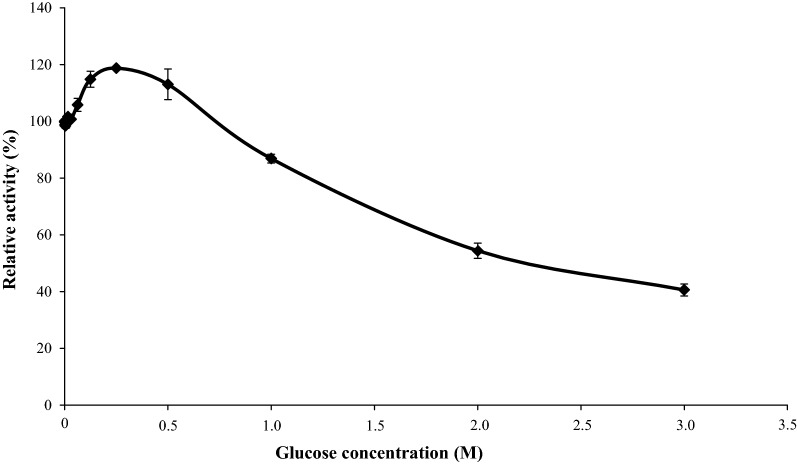
Inhibitory effect of glucose on the activity of BGL-1 in hydrolysis of *p*NPG

Two main features of the inhibition constants of BGL-1, calculated towards glucose, merit special attention: (i) the *K*_*i*_ value was very high (3.78 ± 0.13 M), which to the best of our knowledge, is one of the highest reported until date [18] and (ii) the activity of BGL-1 was stimulated by low concentrations of glucose (Fig. 1). In fact, BGL-1 activity was improved 1.18-fold in the presence of 0.25 M of glucose. At this point, the activity begins to decrease, although it still retained 40% of its initial activity at 3 M glucose (Fig. 1). Regarding its kinetic constants, BGL-1 showed very high efficiency and good affinity on *p*NPG and *o*NPG (Table 1) and it also showed residual activity against *p*-nitrophenyl-β-d-xylopyranoside (5.33 ± 0.28 U/mg). However, its catalytic efficiency (Table 1), against cellooligosaccharides from cellobiose to cellohexaose was low, compared to those reported for the BGLs of the GH3 family characterized in this fungus [14, 15]. Nevertheless, these results agree with those reported for other fungal BGLs from the GH1 family. Besides, an interesting discovery was made when examining the regioselectivity in hydrolysis reactions catalyzed by BGL-1, using cellobiose, sophorose, laminaribiose and gentiobiose as substrates. While the activity over laminaribiose and gentiobiose was negligible (below 10 U/mg), it was considerably high on cellobiose (110.27 ± 4.38 U/mg), but it was fivefold higher on sophorose (535.82 ± 15.83 U/mg). According to these results, BGL-1 could be considered as a versatile β-1,2 BGL, as it is more active on β-1,2 linkages but can also break β-1,4 bonds.

**Table 1:** Kinetic constants of BGL-1 hydrolyzing different substrates

Substrate	*K*_*m*_ (mM)	*k*_cat_ (s^−1^)	*k*_cat_/*K*_*m*_ (mM^−1^·s^−1^)
*p*NPG	3.3 ± 0.7	898.3 ± 23.1	267.3
*o*NPG	2.3 ± 0.6	135.7 ± 5.9	57.5
Cellobiose	20.3 ± 3.4	137.7 ± 3.8	6.7
Cellotriose	19.3 ± 5.4	196.2 ± 6.7	10.1
Cellotetraose	17.6 ± 0.6	276.6 ± 7.2	15.6
Cellopentaose	12.4 ± 0.2	260.4 ± 9.2	20.9
Cellohexaose	9.1 ± 0.6	217.8 ± 2.5	23.7

### Transglycosylation profile and regioselectivity of BGL-1

**Fig. 2:**
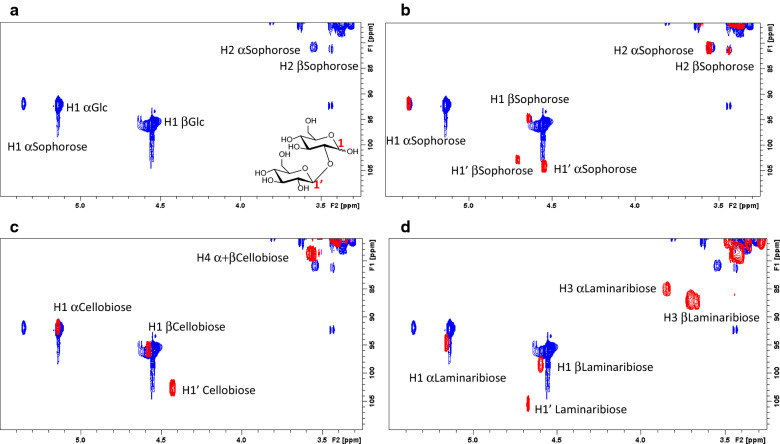
^1^H-^13^C HSQC NMR spectra of the reaction mixture of transglycosylation catalyzed by the native BGL-1 on *p*NPG as substrate (3 mM) in the presence of ^13^C-labelled glucose. **a** Detail of the region of the spectra over ^13^C 75 ppm. The anomeric region is presented for only the signals of labelled glucose, either unreacted or as part of the transglycosylation product. The regiochemistry corresponding to substitution on positon 2 on the labeled glucose is deduced from the superimposition of the spectra of the reaction mixture (in blue) with the spectra of: **b** sophorose (in red). The signals from H1′ of α- and β-sophorose are not observed in the spectrum of the reaction mixture (blue) since the non-reducing residue comes from the unlabeled donor; **c** cellobiose (in red); **d** laminaribiose (in red)

In order to test the transglycosylation capacities of BGL-1, a screening with a variety of potential acceptors, including sugars, sterols, phenolic compounds, or amino acids was performed, according to the methodology previously developed [19, 20]. BGL-1 only showed potential for transglycosylating *p*-nitrophenyl sugar derivatives, like *p*NPG, *p*NPGal, or *p*NPX, which ruled out most of the potential acceptors tested (data not shown). On the other hand, to assess the regioselectivity of the transglycosylation, the products of a model reaction, set up with *p*NPG as donor and ^13^C-labelled glucose as acceptor, were analyzed by Nuclear Magnetic Resonance (NMR). The spectra of the compounds detected in the reaction were compared with those from sophorose, cellobiose and laminaribiose, confirming their coincidence with the sophorose pattern (Fig. 2), and also confirming the β-1,2 selectivity of BGL-1 in transglycosylation.

### Conversion of BGL-1 into glycosynthases by rational design

In order to improve the low efficiency of transglycosylation of BGL-1, a glycosynthase version of the enzyme was developed. The replacement of its catalytic nucleophilic residue, a glutamic acid at position 521, by a glycine (BGL-1-E521G) or a serine (BGL-1-E521S) produced two novel glycosynthases from this enzyme. Both versions of the protein were produced in *P. pastoris* and purified in a unique chromatographic step using the same strategy as for BGL-1. Their activity and efficiency were compared in the transglycosylation of α-GlcF and *p*NPG (10 mM each), proving that both purified glycosynthases produced the expected products. However, the activity of the glycine mutant (BGL-1-E521G) was twofold higher to that of the serine mutant and was selected for further experiments. The study of its kinetic parameters revealed that its affinity for *p*NPG (*K*_m_ 90.14 mM) was higher than for α-GlcF (*K*_m_ 260.86 mM), although the catalytic constant was similar for both substrates (*k*_cat_ 0.11 s^−1^ and 0.08 s^−1^, respectively).

### Transglycosylation of selected acceptors and analysis of the products

The transglycosylation capacities of the BGL-1-E521G mutant were analyzed testing several aryl-glycosides (*p*NPG, *p*NPX, *p*NPGal) and some phenolic compounds (hydroxytyrosol, gallic acid, epigallocatechin gallate (EGCG), and vanillin) as acceptors, and α-GlcF as the donor in every reaction (Additional file 1: Figure S1). In a first approximation, thin layer chromatography (TLC) analysis was used to detect the synthesis of glycosides from the selected acceptors, identifying positive spots for each potential glycoside (Additional file 1: Figure S2). The identity of the expected compounds was confirmed by mass spectrometry (MS), from the coincidence of the molecular mass of the newly-synthesized glucosides with the expected value. All the targets were detected as sodium adducts (Additional file 1: Figure S1). It is interesting to highlight that MS analysis revealed the presence of additional products with higher molecular mass in all reactions. The peaks were identified as saccharides with two (G2), three (G3), four (G4) and five (G5) glucose units, showing that this glycosynthase can also generate oligosaccharides. The presence of non-fluorinated derivatives of these molecules could be due to self-hydrolysis of α-GlcF or the fluorinated derivatives during overnight reactions. Besides, considering that the native BGL-1 hydrolyzes *p*NPX, and the capacity of BGL-1-E521G to use *p*NPX as transglycosylation acceptor, the potential use of d-xylosyl fluoride as donor of the reaction should not be discarded, which may expand the applications of the enzyme.

The reactions rendering the most intense signals from products in TLC and MS (corresponding to *p*NPG, *p*NPX, *p*NPGal, and EGCG as acceptors) were analyzed by High Performance Liquid Chromatography (HPLC) to determine the transglycosylation yields and to purify the main products for further NMR analysis. The conversions were 73.5% for *p*NPG, 89.8% for *p*NPX, 36.6% for *p*NPGal, and, more interestingly, the glucoside of EGCG was obtained with a very significant yield of 48.8% (chromatograms not shown). Besides, a second product from EGCG, that eluted before the main product during the purification procedure, was also isolated.

### Products characterization by NMR

**Fig. 3:**
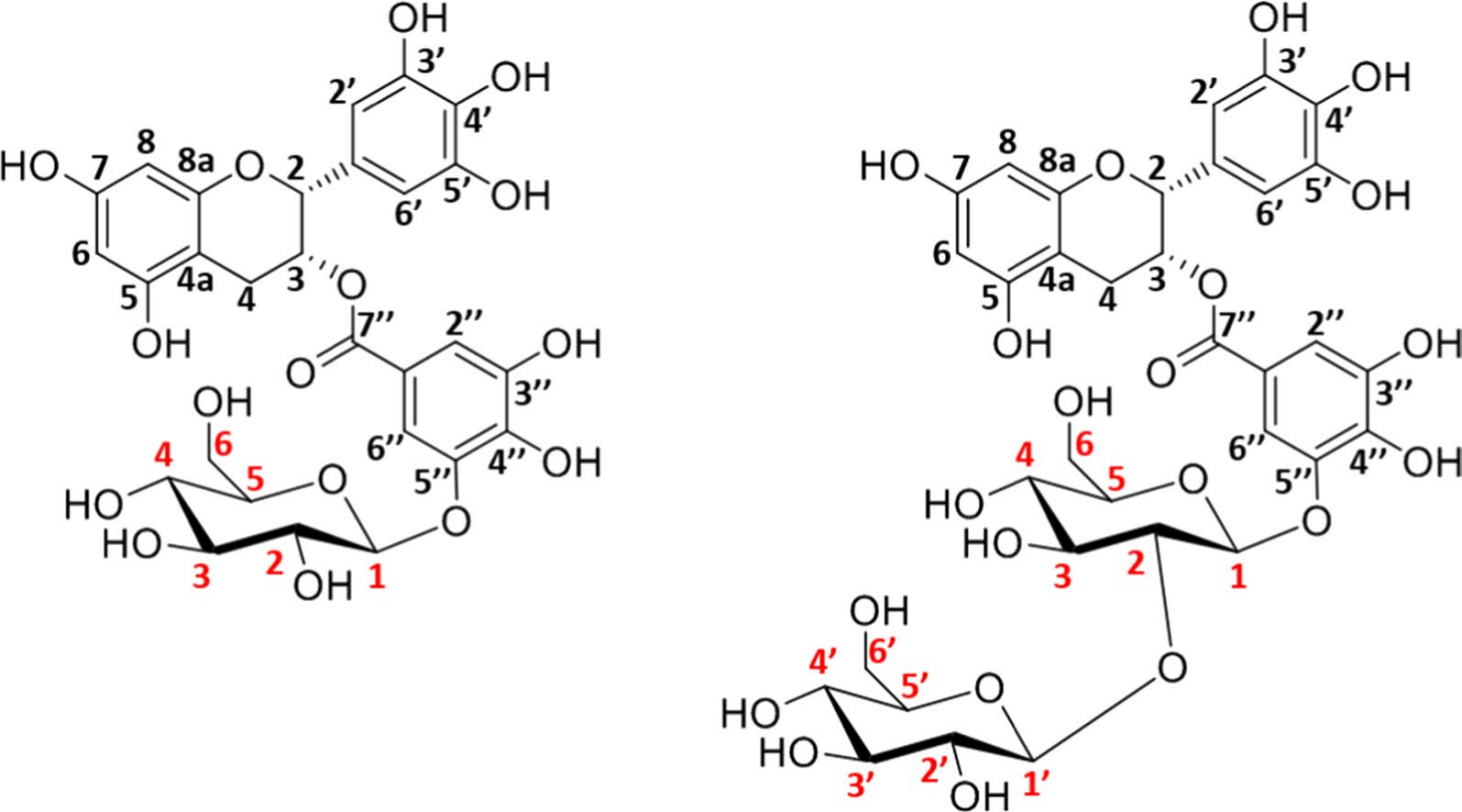
Structures, deduced from the NMR analysis, of the two glucosides produced by transglycosylation of EGCG with the synthase BGL-1-E521G. Every C atom in the molecules is numbered to clarify the identification of the signals

**Fig. 4:**
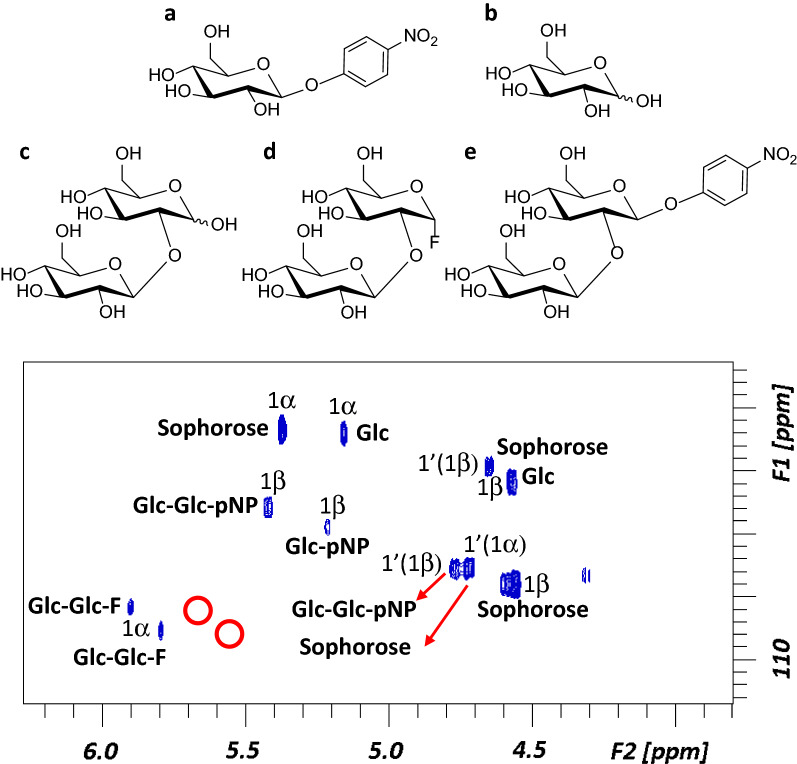
(1) Structures of *p*NPG (**a**), Glc (**b**), sophorose (**c**), sophorose-F (**d**) and sophorose-*p*NP (**e**). (2) Anomeric region of the ^1^H-^13^C HSQC spectrum of a transglycosylation reaction mixture with pNPG as acceptor and αGlc-F as donor, catalyzed by BGL-1-E521G.The peaks are labeled with their corresponding assignment. Red circles indicate where the signals corresponding Glc-F, which has been fully consumed, should appear

Once purified, the glucosides of EGCG, *p*NPG, *p*NPX and *p*NPGal were analyzed by ^1^H and ^13^C-NMR, in order to confirm their structure and assign their regiochemistry. The HMBC (Heteronuclear Multiple Bond Correlation) spectra of the two EGCG-glucose derivatives (Additional file 1: Figures S4 and S5) showed a correlation between the anomeric position of glucose and the *meta*-carbon (3″/5″) of the gallate aromatic ring, indicating the position of the linkage between the phenolic and the sugar moieties in these glycoconjugates (Fig. 3). In addition, the second sugar unit in the EGCG-disaccharide is attached to the *O*-2 of the first one through a β-linkage, as deduced from the value of the coupling constant of the anomeric proton (7 Hz) and in accordance with the spectral assignment (Table 2). The results from the NMR analysis of the three *p*NP derivatives and their NMR spectra are shown in Additional file 1: Figures S6, S7 and S8. All of them indicate the regioselectivity of the glycosynthase that specifically forms *p*NP-disaccharides, incorporating the second sugar unit through a β-1,2 linkage. Finally, the different molecular species produced in a crude transglycosylation reaction mixture of α-GlcF (donor) and *p*NPG (acceptor) were also analyzed by NMR. Interestingly, this sample showed significant heterogeneity as observed in the anomeric region of the ^1^H-^13^C HSQC (Heteronuclear Single-Quantum Correlation) spectrum of the mixture (Fig. 4). The presence of some unreacted acceptor *p*NPG-glucose but not starting donor α-GlcF, was confirmed. Unexpectedly, signals tentatively assigned to α-F-sophorose were observed indicating that the α-GlcF itself with the α configuration could fit in the acceptor site. Besides, free glucose was also identified, confirming the auto-hydrolysis of the fluorinated substrate during the reaction. To note, this free glucose also worked as acceptor of α-GlcF, as deduced from the presence of sophorose among the reaction products. Again, the newly-synthetized disaccharides were linked by β-1,2 bonds, confirming the total regioselectivity of the synthase, which follows the same behavior than BGL-1, just being able to transglycosylate in this position.

**Table 2:** Chemical shifts for EGCG-glucose and EGCG-sophorose

EGCG-glucose			EGCG-sophorose		
	^1^H	^13^C		^1^H	^13^C
2	5.08	77.06	2	5.03	77.05
3	5.60	68.89	3	5.57	68.71
4	2.86	24.91	4	2.84	24.86
	3.01			2.97	
4a	–	99.04	4a	–	99.00
5	–	155.18	5	–	155.34
6	6.08	95.72	6	6.06	96.01
7	–	155.17	7	–	155.34
8	6.08	95.72	8	6.06	96.01
8a	–	155.18	8a	–	155.21
1′	–	106.33	1′	–	106.39
2′	6.48	106.45	2′	6.47	106.40
3′	–	145.42	3′	–	145.51
4′	–	132.10	4′	–	132.62
5′	–	145.44	5′	–	145.65
6′	6.48	106.45	6′	6.47	106.40
1′′	–	120.59	1′′	–	120.58
2′′/6′′	7.09	108.61	2′′/6′′	7.06	108.61
3′′	–	144.30	3′′	–	144.59
4′′	–	139.73	4′′	–	139.90
5′′	–	144.27	5′′	–	144.59
6′′/2′′	7.06	112.27	6′′/2′′	7.05	112.18
7′′	–	166.50	7′′	–	166.43
1 Glc	4.97	100.33	1 Glc	5.09	99.22
2 Glc	3.53	72.60	2 Glc	3.78	80.98
3 Glc	3.54	75.32	3 Glc	3.69	75.39
4 Glc	3.49	68.65	4 Glc	3.53	68.29
5 Glc	3.22	75.97	5 Glc	3.21	75.63
6 Glc	3.41	59.73	6 Glc	3.41	59.83
	3.66			3.66	
			1′ Glc	4.77	102.77
			2′ Glc	3.25	73.85
			3′ Glc	3.43	75.70
			4′ Glc	3.36	69.36
			5′ Glc	3.29	76.09
			6′ Glc	3.25	60.33
				3.53

## Discussion

*Talaromyces amestolkiae* has recently been postulated as a very interesting option for producing enzymatic cocktails rich in BGLs [13]. Two BGLs from this fungus have already been characterized and classified as members of the GH3 family [14, 15], which is usually considered the one encompassing the BGLs with better catalytic efficiency on cellooligosaccharides. However, as already mentioned, they are fairly low glucotolerant [21], and most of the glucose-tolerant BGLs characterized so far belong to the GH1 family [17]. For this reason, the identification of potential glucotolerant GH1 BGLs in the genome and proteome of *T. amestolkiae* could increase the value of its cellulolytic system. The recombinant enzyme was produced in the methylotrophic yeast *P. pastoris*, successfully used as host for the expression of eukaryotic genes, including those from other BGLs from this fungus [14, 15]. Some additional advantages of this organism are its ability to synthesize high levels of correctly-folded proteins, and to perform complex post-translational modifications [16]. The production level of 75 U/mL is among the highest for BGLs reported in the literature. To the best of our knowledge, this value is only surpassed by those found for the recombinant PtBglu3 from *Paecilomyces thermophila* [22], and bgl3A, from *Talaromyces leycettanus* [23]. In general, most of the recombinant strains producing BGLs reach an activity between 1 and 50 U/mL, which highlights the expression levels of BGL-1 [5, 14, 15, 24–27]. It is important to emphasize that the activity determined for this recombinant BGL-1 was 35-fold higher than the total β-glucosidase activity detected in cultures of *T. amestolkiae* [14], which corresponds to the sum of the activities of BGL-1, BGL-2 and BGL-3.

An interesting feature of BGL-1 that may postulate this enzyme as a candidate for industrial processes performed at high glucose concentrations is its ability to retain an impressive catalysis capacity even at high levels of this product. Regarding glucotolerance, it has been observed in several GH1 [21] and in a few GH3 β-glucosidases [5]. But the behavior responsible of the glucose-induced activity stimulation remains unknown. Its relation to an allosteric effect triggered by the binding of glucose to some part of the protein, or to an increased hydrolysis rate upon transglycosylation has been suggested [28]. However, as already explained, the high catalytic efficiency of GH3 β-glucosidases makes them the enzymes of choice for cellulose saccharification, even presenting *Ki* values < 0.1 M, while the glucotolerant GH1 β-glucosidases usually have lower catalytic efficiency over cellooligosaccharides [3, 21]. According to their substrate preferences, β-glucosidases can be classified into three groups: cellobiases, with high substrate specificity towards cellooligosaccharides, aryl-β-glucosidases, very specific for synthetic substrates such as *p*-nitrophenyl-β-d-glucopyranoside (*p*NPG), and β-glucosidases with broad substrate specificity, that combine both activities [17]. For BGL-1, the *k*_*cat*_ values calculated for hydrolysis of cellooligosaccharides were relatively good, but its *K*_*m*_ values were poor when compared with those observed for BGLs from the GH3 family [5, 14, 15, 23]. This result confirms the low affinity of BGL-1 for these substrates, similarly to other GH1 β-glucosidases [18, 21, 29–32], which could limit its applicability in hydrolytic processes despite its enormous glucotolerance. However, BGL-1 could be useful if added to efficient GH3 BGL mixtures, for its ability to extend saccharification once they are inactivated by glucose.

Another feature of BGL-1 was its higher hydrolytic activity over sophorose than on cellobiose. This behavior, although initially unexpected, seems to be common in GH1 enzymes, as it has recently been reported by Heins et al. [33]. In this study, more than 170 GH1 proteins were analyzed by means of a high-throughput screening approach, corroborating a common pattern of hydrolysis of β-1,2 bonds. Nevertheless, few of these β-1,2-glycosidases have been completely characterized so far. For example, some glucanases and glucosidases induced by β-1,2-glucan have been discovered in *Acremonium sp*., a filamentous anamorphic fungus [34], but their amino acid sequences have not been determined, which precludes their comparison with the BGL-1 from *T. amestolkiae*. Recently, a BGL with activity on β-1,2 bonds was reported in the bacterium *Listeria innocua.* It was not able to hydrolyze cellobiose, and its physiological role was not completely established, although the authors suggest that it was involved in β-1,2-glucan dissimilation [35]. On the other hand, it is well known that sophorose is the most powerful inducer of cellulases in *T. reesei* [36]. A recent report describes the production of this disaccharide by transglycosylation catalyzed by intracellular BGLs of this fungus, and its potential role in the regulation system of cellulase induction [37]. Therefore, taking into account that BGL-1 is produced by *T. amestolkiae* in all conditions assayed [13], and that this enzyme can hydrolyze and synthesize β-1,2 bonds, its physiological role could be related to the regulation of the induction of the cellulolytic system in this organism.

Regarding transglycosylation, the native BGL-1 showed a very poor profile of potential acceptors, in contrast with the good results obtained with the GH3 BGLs of *T. amestolkiae* [20]. Historically, enzyme engineering has been successfully implemented to enhance the transglycosylation activity of glycosidases and, simultaneously, attenuate hydrolysis. This kind of enzymes were first reported by Withers and coworkers [9], who noticed that a mutated glycoside hydrolase lacking its catalytic nucleophile can use activated glycosyl fluoride donors with the opposite anomeric configuration for synthesizing glycosides, without hydrolyzing the products. This approach has been successfully applied to convert glycosidases from the GH1 family into glycosynthases, from GH36 in galactosynthases, or from GH29 in fucosynthases [10]. Two variants of BGL-1 from *T. amest*olkiae, BGL-1-E521G and BGL-1-E521S were produced, purified, and compared. These mutations have been shown to be much more efficient in the synthesis of oligosaccharides and *p*-nitrophenyl derivatives than the traditional alanine replacement for creating glycosynthases [8].The BGL-1-E521G variant displayed higher activity than the serine mutant, and this finding agrees with published data suggesting that the rigid serine side-chain could make the departure of the fluorine more difficult, which is instead stimulated in the glycine mutants [38–40]. Other authors justify this different behavior between the mutants considering that the lack of a side-chain in glycine resulted in a reduced steric hindrance compared with the alanine or serine mutants [41]. The kinetic constants for BGL-1-E521G exhibited slightly worst performance in *K*_*m*_ than reported for a xylosynthase [42], but were similar in terms of *k*_*cat*_, confirming that the obtained glycosynthase could be a valuable starting point for optimizing biocatalytic transglycosylation reactions.

Finally, with respect to the potential acceptors of transglycosylation for the novel glycosynthase BGL-1-E521. *p*NP-sugars have been frequently used as preferential transglycosylation acceptors for glycosynthases, generating a variety of products, from the expected *p*NP-disaccharides, to *p*NP-oligosaccharides of different length and regioselectivity [8]. As most of the glycosynthases reported until date, BGL-1-E521G generated glycoconjugates of *p*NP-sugars [11]. Besides these, phenolic acceptors are very interesting transglycosylation targets, because value-added glycosides can be obtained from this type of compounds. Some phenols have shown a variety of beneficial properties related to human health, and have been reported to confer cardiovascular protection, and possess a positive effect in neurodegenerative diseases and cancer [43]. One of the main disadvantages of these substances is their low bioavailability that can be enhanced through glycosylation, which can increase the solubility of the aglycon. In this context, various studies have demonstrated the interesting properties of hydroxytyrosol, vanillin and gallic acid [44–46], and EGCG has recently attracted attention as a potential therapeutic agent [47, 48], also in its glycosylated forms [49]. BGL-1-E521G was able to generate glycosides of all these molecules, demonstrating that this enzyme is a versatile tool for the transglycosylation of several bioactive compounds. It should be noted that the conversion rate of 48.8% in transglycosylation of EGCG is among the best reported in the literature, although yields of 58% [50] and 91% [51], have also been reported for the enzymatic synthesis of the same compound using a cyclodextrin glucanotransferase and a dextransucrase, respectively. Optimization of EGCG transglycosylation catalyzed by BGL-1-E521G will be explored in future works in order to enhance the product yields.

## Conclusions

BGL-1, the novel GH1 β-glucosidase from the ascomycete *T. amestolkiae,* has been heterologously produced in *P. pastoris*. The recombinant enzyme was easily purified with good yield (80%) and then characterized and evaluated in hydrolysis and transglycosylation reactions. BGL-1 was remarkably glucotolerant, as deduced from its inhibition constant (3.78 M), although its low catalytic efficiency in the hydrolysis of cellooligosaccharides may limit its applications in saccharification. This enzyme, tested as catalyst for transglycosylation of a wide array of compounds, showed a poor profile of potential acceptors although with high β-1,2 selectivity. The conversion of BGL-1 into a glycosynthase, by protein engineering, generated the novel biocatalyst (BGL-1-E521G), which turned out to be a versatile tool for regioselective β-1,2 transglycosylation. Besides, it catalyzed the transfer of glucose molecules not only to *p*-nitrophenyl sugars, but also to interesting phenolic acceptors such as EGCG, a compound with many potential medical applications. These findings postulate this mutant as a potential candidate to be used in biotechnological processes devoted to the synthesis of bioactive glycosides.

## Materials and methods

### Microorganism and culture conditions

For maintenance, the fungus *T. amestolkiae* A795, deposited in the Centro de Investigaciones Biológicas (Madrid, Spain), was cultured in PDA (potato dextrose agar) plates at 28 °C. *T. amestolkiae* was then cultured in Mandels medium, as reported before [15], with 1% of Avicel as carbon source, for RNA extraction.

*Escherichia coli* DH5α (Invitrogen) was used for plasmid propagation. Cells were grown at 37 °C, in LB medium (10 g/L tryptone, 5 g/L yeast extract, 10 g/L NaCl, and 15 g/L agar) containing 25 mg/L zeocin for selection of resistant colonies.

The heterologous expression of ΒGL-1 was performed using *P. pastoris* X33 strain (Invitrogen), previously grown in YPD medium plates (10 g/L yeast extract, 20 g/L peptone, 20 g/L glucose and 10 g/L of agar). The positive clones were screened in YPD containing 100 mg/L of zeocin as selection marker, and cultured for 2–5 days at 28 °C. Recombinant protein was produced in YEPS medium (20 g/L peptone, 10 g/L yeast extract, 10 g/L sorbitol, and 100 mM potassium phosphate buffer, pH 6), with daily addition of 10 mL/L of methanol as inducer. Cultures were incubated for 9 days, at 28 °C and 250 rpm, taking samples daily to measure BGL-1 production. All experiments were performed in triplicate.

### Nucleic acid isolation, enzyme mutagenesis and cloning in *Pichia pastoris*

The DNA sequence of *bgl*-*1* was identified by performing a TBLASTN against the set of predicted proteins of *T. amestolkiae*, obtained in a previous work [13]. The gene sequences returned were used to run a local BLASTN against the assembled genome [13]. The presence of a signal peptide in the BGL-1 protein sequence was examined using the SignalP server [52]. RNA was extracted from 7-day old *T. amestolkiae* cultures growing in 1% of Avicel using Trizol reagent [53]. The isolated transcripts were converted to cDNA by RT-PCR using the Superscript II Reverse Transcriptase according to the manufacturer’s instructions. PCR amplifications were performed in a thermocycler Mastercycler pro S (Eppendorf). The primers were designed based on the nucleotide sequence of the *bgl*-*1* gene identified in *T. amestolkiae* genome (GenBank accession no. KM393204.1), excluding the region corresponding to the signal peptide. Restriction sites for *Xho*I and *Not*I were included in the forward and reverse primers respectively (BG1FWXHOI: 5′-ATCTCGAGAAAAGACAAGAGGTGTACATCACGACT-3′, and BG1RVNOTI: 5′-ATGCGGCCGCATATCCCAGCCCATTCCTCGC-3′). The PCR protocol was: first, a denaturation step at 95 °C for 5 min, followed by 36 cycles of amplification: denaturation at 95 °C for 45 s, primer annealing at 55 °C for 45 s, and elongation at 72 °C for 2 min. A final extension step at 72 °C for 10 min was also carried out. The PCR product obtained in the last step was introduced in the pPICzα expression vector (Invitrogen), and it was used to transform *P. pastoris* X-33 strain. Before transformation, the vector was linearized with *SacI* (New England Biolabs). The lithium chloride method was used for transformation according to the manufacturer’s instructions. Transformed colonies were grown on YPD medium plates with zeocin as selection marker. Positive clones were screened with 4-methylumbelliferyl β-d-glucopyranoside as described in Méndez-Líter et al. [14].

### Conversion of BGL-1 into the two glycosynthase variants

The plasmid pPICzα containing *bgl*-*1* gene was used to generate two new versions of the protein by directed mutagenesis, replacing the glutamic acid 521 by a glycine or a serine. The identification of the catalytic amino acids of BGL-1 was performed by alignment using clustal omega (see Additional file 1: Figure S3), with the BGLs sequences of the bacterium *Clostridium cellulovorans*, the fungus *Trichoderma reesei* and the termite *Neotermes koshunensis*, in which the nucleophilic amino acid were previously detected by crystallographic methods [54]. For the mutagenic PCR, the Expand™ Long Template PCR System (Roche) was used as described by the manufacturer. Primers BG1sfwSer (CCCTCGTCCTCAGCTCATTCGGTTTTCCCGTCTAC), BG1sRvSer (GTAGACGGGAAAACCGAATGAGCTGAGGACGAGGG), BG1sfwGly (CCCTCGTCCTCAGCGGATTCGGTTTTCCCGTCTAC) and BG1sRvGly (GTAGACGGGAAAACCGAATCCGCTGAGGACGAGGG) were used for serine and glycine replacements, respectively. After PCR reaction, the product was digested by *DpnI* (New England Biolabs), in order to hydrolyze the methylated parental. Both new vectors were cloned into *P. pastoris* with the same method used previously.

### Production and purification of BGL-1 and BGL-1 glycosynthase variants

The selected positive *P. pastoris* clones were grown overnight in 250 mL flasks with 50 mL of YPD medium at 28 °C and 250 rpm to obtain the respective preinocula. Then, they were used for recombinant protein production in 2-L flasks with 400 mL of YEPS medium. Cultures were incubated at 28 °C and 250 rpm for 9 days with daily addition of 10 mL/L methanol. For BGL-1 and its glycosynthase variants purification, 1 L of 9 day-old cultures was harvested and centrifuged at 10,000×*g* and 4 °C for 20 min. The supernatant was concentrated to 20 mL and dialyzed against 10 mM phosphate buffer (pH 6.0). BGL-1 was purified in a single chromatographic step using an FPLC system (Äkta), with a 5 mL QFF HiTrap cartridge (GE Healthcare) equilibrated with phosphate buffer 10 mM pH 6.0. Elution of the bound proteins was carried out by applying a 25 min-linear gradient from 0 to 0.3 M of NaCl in phosphate buffer pH 6.0 10 mM, at 2 mL/min. The column was then washed with 10 mL of 1 M NaCl in phosphate buffer pH 6.0 10 mM and re-equilibrated using 10 mL of the starting buffer. Fractions with β-glucosidase activity were dialyzed and concentrated.

### Protein quantification, enzyme assays and substrate specificity

Protein concentration of the purified proteins was determined measuring 280 nm absorbance in a Nanodrop spectrophotometer (Thermo Fisher Scientific).

The β-glucosidase standard reaction was performed in a heating block at 60 °C and 1200 rpm, using 3 mM *p*-nitrophenyl-β-d-glucopyranoside (*p*NPG, Sigma) in sodium acetate buffer 50 mM, pH 4.0. The reaction was stopped after 10 min by adding 1.4% (w/v) Na_2_CO_3_, and the release of *p*-nitrophenol (*p*NP) was measured in a spectrophotometer at 410 nm. One BGL activity unit was defined as the amount of enzyme capable of releasing 1 μmol of *p*NP per min (the molar extinction coefficient of *p*NP is 15,200 M^−1^·cm^−1^).

Glucose tolerance was determined by measuring β-glucosidase activity in standard conditions but in the presence of 0.1 mM to 3 M glucose concentrations. Activity in reactions without glucose were considered as 100%. For *K*_*i*_ determinations, the concentrations of glucose used were 1, 1.25, and 1.5 M (Additional file 1: Figure S9).

To prevent the activity loss when working with low enzyme concentrations, all enzymatic assays included 0.1% BSA, a protein that does not affect the catalytic activity of BGL-1 [55]. The kinetic constants of the purified BGL-1 were determined against *p*NPG over a range of concentrations from 10 μM to 5 mM, *o*-nitrophenyl-β-d-glucopyranoside (*o*NPG, 40 μM to 20 mM), cellobiose (80 μM to 40 mM), cellotriose (80 μM to 40 mM), cellotetraose (80 μM 40 mM), cellopentaose (40 μM to 20 mM), and cellohexaose (20 μM to 10 mM). The *K*_*m*_ and *V*_*max*_ parameters were calculated using SigmaPlot (Stat-Ease). These reactions were quantified by measuring the glucose released using the Glucose-TR commercial kit (Spinreact) according to the manufacturer’s instructions. All reactions were carried out in sodium acetate 50 mM, pH 4.0, in a heating block (1200 rpm), at 60 °C, during 10 min. Then, the reactions were stopped by heating them at 100 °C for 5 min.

Kinetic parameters were calculated for two transglycosylation experiments catalyzed by the BGL-1-E521G variant with D-glucosyl fluoride (α-GlcF) and *p*NPG as substrates. Each substrate was used in one experiment at a fixed concentration of 10 mM, and with varying concentrations in the other. When *p*NPG was examined, concentrations ranged between 500 and 12.5 mM. When calculating kinetic constants for α-GlcF, it was used in a range from 25 mM to 1 M.

BGL-1 activity towards cellobiose, sophorose, laminaribiose, and gentiobiose, was determined using 10 mM of the disaccharides in sodium acetate buffer 100 mM, pH 4.0, with the appropriate amount of enzyme. Reactions were performed for 10 min at 60 °C and 1200 rpm, and quantified by measuring the glucose released using the Glucose-TR commercial kit (Spinreact).

### Physicochemical properties

The molecular mass of the native BGL-1 was determined by MALDI-TOF [15]. The isoelectric point (pI) was determined by isoelectrofocusing (IEF), after revealing the gel with 4-methylumbelliferyl-β-d-glucopyranoside [14]. The optimal pH was evaluated using *p*NPG as substrate at 60 °C, in a in 10 min-BGL standard reaction in Britton-Robinson buffer (100 mM) in a range from 2 to 10. The optimal temperature was assayed using also standard conditions but varying the temperature from 30 to 70 °C.

### Screening for transglycosylation acceptors of BGL-1

Potential transglycosylation acceptors for the wild type BGL-1 were searched against a library of 70 compound through a preliminary screening carrying out recovery inhibition assays, as described in a previous work [20]. Those that produced reaction rates higher than the controls without acceptor were considered potential hits for transglycosylation. The complete list of the tested acceptors is: 1-butanol, 1-heptanol, 1-propanol, 2,4-dinitrophenol, 2,6-dihydroxynaphthalene, 2-butanol, 2-mercaptoethanol, 2-nitrophenyl β-d-glucopyranoside, 2-propanol, 3,3-diphenyl propanol, 4-cresol, 4-hydroxybenzyl alcohol, 4-methylumbilliferyl β-d-xylopyranoside, 4-nitrophenol, 4-nitrophenyl α-arabinopyranoside, 4-nitrophenyl α-d-glucopyranoside, 4-nitrophenyl α-d-rhamnopyranoside, 4-nitrophenyl β-d-fucopyranoside, 4-nitrophenyl β-d-galactopyranoside, 4-nitrophenyl β-d-glucopyranoside, 4-nitrophenyl β-d-xylopyranoside, l-arabinose, arabitol, ascorbic acid, catechol, cellobiose, cinnamyl alcohol, cyclohexanol, dulcitol, EGCG, ergosterol, ethanol, eugenol, ferulic acid, d-fructose, d-galactose, gallic acid, gentiobiose, d-glucose, glycerol, guaiacol, hydroquinone, hydroxytyrosol, myo-inositol, lactose, maltose, mannitol, d-mannose, melibiose, menthol, methanol, naphthol, phenol, propargyl alcohol, quercetin, raffinose, resveratrol, d-ribose, l-serine, sorbitol, sorbose, sucrose, l-threonine, l-trehalose, l-tyrosine, vanillyl alcohol, xylitol, d-xylose, α-tocopherol, β-sitosterol.

### Transglycosylation reactions catalyzed by the glycosynthases. Analysis of the products

In these reactions, α-GlcF was prepared as previously described [56], and used as donor in every reaction. The efficiency of both glycosynthase variants (BGL-1-E521G and BGL-1-E521S) was first compared in reactions containing 10 mM *p*NPG, 10 mM α-GlcF, 0.4 mg/mL of the corresponding mutant enzyme, and 50 mM acetate buffer pH 4. The reaction mixture was incubated at room temperature for 16 h at 500 rpm, and then analyzed by HPLC as explained below.

Other potential acceptors tested were: *p*-nitrophenyl-β-d-xylopyranoside (*p*NPX), *p*-nitrophenyl-β-d-galactopyranoside (*p*NPGal), vanillin, hydroxytyrosol, gallic acid, and EGCG. The standard transglycosylation reaction contained 20 mg/mL of α-GlcF, 5 mg/mL of each acceptor and 1 mg/mL of BGL-1-E521G in acetate buffer 50 mM, pH 4, with 0.1% of BSA, and it was performed at room temperature for 16 h at 500 rpm. The synthesis of glycosides was first checked by TLC in silica gel G/UV254 polyester sheets provided by Macherey–Nagel. The running solution was contained ethyl acetate/methanol/water in 10:2:1 (v/v) proportions. Substrates and glucosides were detected under 254 nm UV light.

Positive hits by TLC were also analyzed by mass spectrometry in a HCT Ultra ion trap, to identify the expected glycosylated products. 10 μL of the samples were introduced by direct infusion and ionized using electrospray ionization-mass spectrometry (ESI–MS) with methanol as ionizing phase in the positive reflector mode. After analysis, data were processed with the Masshunter Data Acquisition B.05.01 and Masshunter Qualitative Analysis B.07.00 software (Agilent Technologies). All the expected products were detected as sodium adducts of the molecules.

Finally, we decided to purify the EGCG, and the *p*NP-derivatives glycosides by HPLC, in an Agilent 1200 series LC instrument equipped with a ZORBAX Eclipse plus C18 column (Agilent). The column was first equilibrated in a mix of acetonitrile (ACN) and H_2_O with 0.1% acetic acid, with a flow of 2 mL/min, and the reaction products were separated isocratically in 8 min. The glycosides of *p*NPG and *p*NPGal, were purified with a proportion of 14:86 (v/v) ACN:H_2_O. For the *p*NPX glycoside this proportion changed to 20:80 (v/v) ACN:H_2_O and for the EGCG products it was 13:87 (v/v) ACN:H_2_O. In all cases, after isocratic elution, the column was washed for 3 min with 95:5 ACN:H_2_O, and the system was finally re-equilibrated to the initial conditions for 4 min. Every product peak was detected by monitoring the absorbance at 270 nm. The quantification of the peaks was done by comparing with a calibration curve of each non glycosylated parental. The fractions containing the glycosides were collected to be further analyzed by NMR to determine their structure.

The reactions conducted to determine the kinetic parameters of BGL-1-E521G were also quantified using the isocratic HPLC method as described above for the glycosides of *p*NPG and *p*NPGal.

### Nuclear magnetic resonance

The structure and regiochemistry of the purified glucosides of EGCG, *p*NPG, *p*NPX and *p*NPGal synthesized by BGL-1-E521G was elucidated by NMR. The samples for the NMR analysis were prepared by dissolving the purified compounds in 500 μL of deuterated water (D_2_O). NMR spectra were acquired at 298 K, using a Bruker AVANCE 600 MHz spectrometer equipped with a cryogenic probe. 1D ^1^H NMR spectra, ^1^H-^13^C HSQC and HMBC experiments were acquired to assign all NMR signals. For 1D ^1^H, ^1^H-^13^C HSQC, and HMBC experiments, the zg, zgpr, hsqcedetgp, and hmbcgpndqf sequences were employed. A standard transglycosylation reaction (described before), without purification of the products, was also submitted to NMR analysis, in order to determine all the species that could be generated by the glycosynthase.

Besides, to determine the regioselectivity of the wild type enzyme, a reaction using the native BGL-1 was performed. It was composed of 3 mM of *p*NPG as donor and ^13^C-labelled glucose as acceptor, in 50 mM acetate buffer pH 4. The reaction was set directly in the NMR at room temperature.

## Supplementary information

**Additional file 1: Figure S1.** Molecular structure of the acceptors selected for transglycosylation and ESI–MS data of glycosides obtained by transglycosylation **Figure S2.** Thin layer chromatography of the different compounds in transglycosylation reactions using BGL-1-E521G. **Figure S3.** Alignment of BGL-1 amino acid sequence. **Figure S4.** NMR Spectra of the EGCG glycoside. **Figure S5.** NMR Spectra of the EGCG plus sophorose. **Figure S6.** NMR Spectra and data of the Glucose-Glucose-*p*NP derivative. **Figure S7.** NMR Spectra and data of the Glucose-Galactose-*p*NP derivative. **Figure S8.** NMR Spectra and data of the Glucose-Xylose-*p*NP derivative. **Figure S9.** Data used for kinetic calculations of inhibition constant by glucose of BGL-1.

## Data Availability

*T. amestolkiae* whole genome shotgun project is deposited at DDBJ/ENA/GenBank under the accession number MIKG00000000. BGL-1 sequence is deposited in GenBank under the accession number KM393204.1.
