# Supplementary material for: A glucotolerant β-glucosidase from the fungus Talaromyces amestolkiae and its conversion into a glycosynthase for glycosylation of phenolic compounds
Source: Microb Cell Fact. 2020 Jun 10;19:127. doi: 10.1186/s12934-020-01386-1 (PMC7288487; doi:10.1186/s12934-020-01386-1)
Supplement: Supplementary file 1 — Additional file 1: Figure S1. Molecular structure of the acceptors selected for transglycosylation and ESI–MS data of glycosides obtained by transglycosylation Figure S2. Thin layer chromatography of the different compounds in transglycosylation reactions using BGL-1-E521G. Figure S3. Alignment of BGL-1 amino acid sequence. Figure S4. NMR Spectra of the EGCG glycoside. Figure S5. NMR Spectra of the EGCG plus sophorose. Figure S6. NMR Spectra and data of the Glucose-Glucose-pNP derivative. Figure S7. NMR Spectra and data of the Glucose-Galactose-pNP derivative. Figure S8. NMR Spectra and data of the Glucose-Xylose-pNP derivative. Figure S9. Data used for kinetic calculations of inhibition constant by glucose of BGL-1. [file 12934_2020_1386_MOESM1_ESM.docx]

## SUPPLEMENTARY INFORMATION


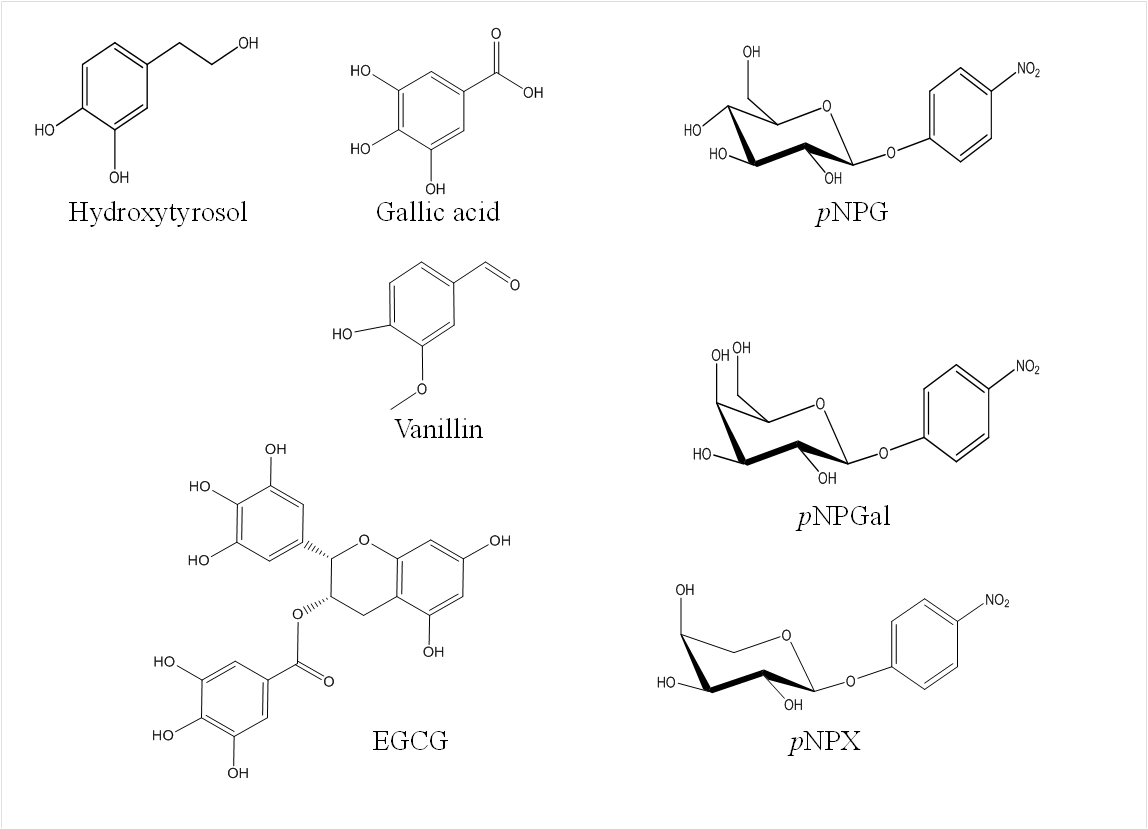


| **Glycoside** |  | **Intensity** | **m/z** |
| --- | --- | --- | --- |
| EGCG-glucose |  | 533662 | 643.2 |
| Vanillin-glucose |  | 43977 | 337.1 |
| Hydroxytyrosol- glucose |  | 22386 | 339.1 |
| Gallic acid-glucose |  | 48294 | 355.1 |
| *p*NPX-glucose |  | 307637 | 456.1 |
| *p*NPGal-glucose |  | 569165 | 486.1 |
| *p*NPG-glucose |  | 430001 | 486.2 |
| G2 |  | 449035 | 365.1 |
| G3 |  | 428105 | 527.2 |
| G4 |  | 287540 | 689.3 |
| G5 |  | 59761 | 851.3 |

**Figure S1. Top:** Molecular structure of the acceptors selected for transglycosylation catalysed by the glucosynthase BGL-1-E521G. **Bottom:** ESI-MS data of the glycosides obtained by transglycosylation catalyzed by BGL-1-E521G. All the glycosides were detected as Na^+^ adducts.


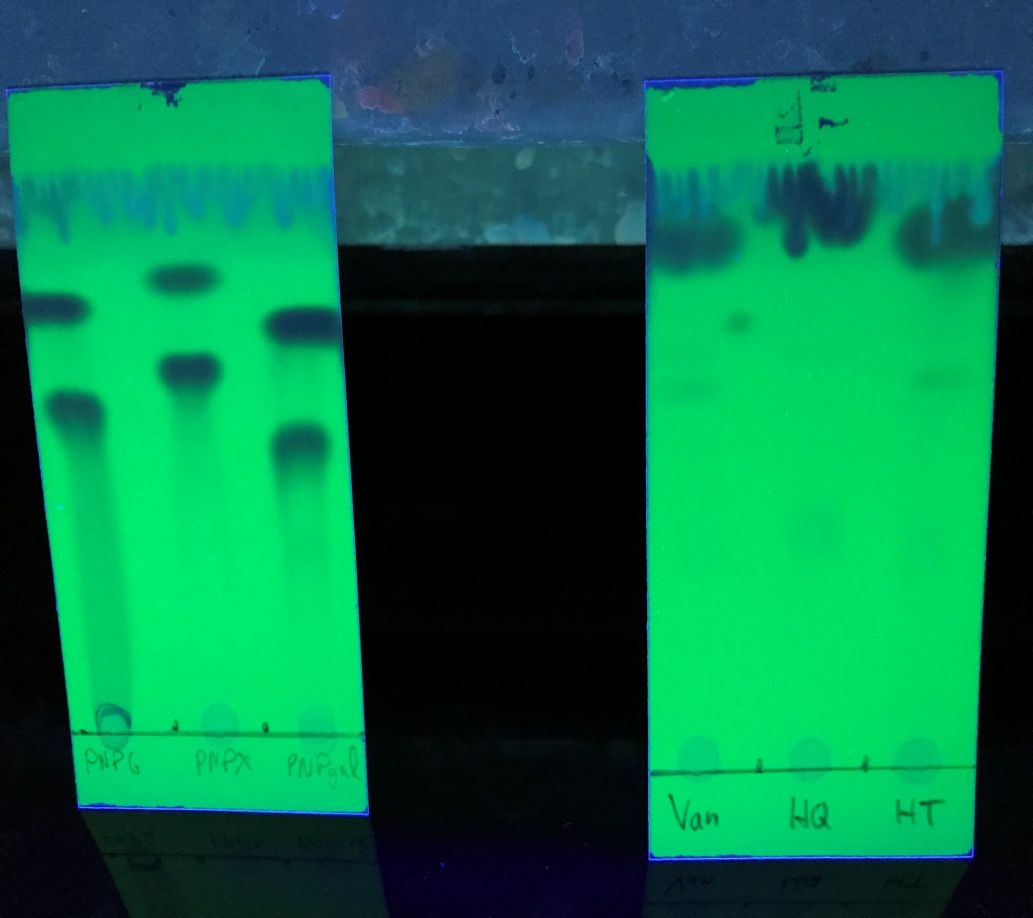


**B**


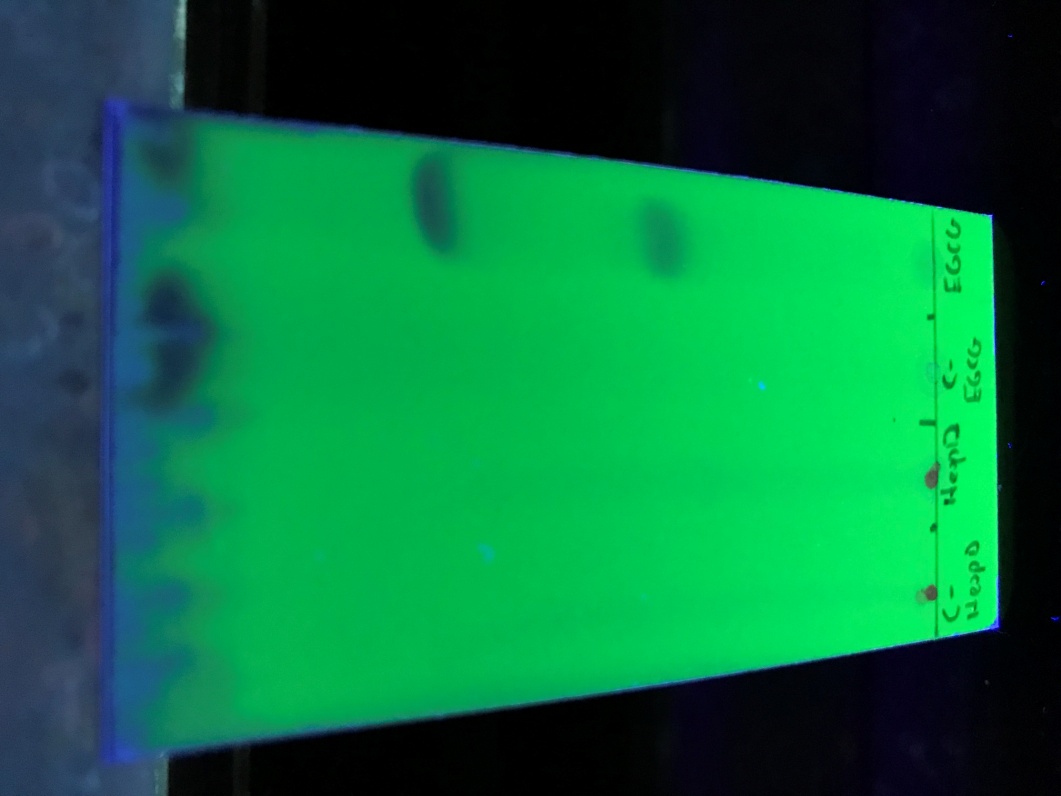


**D**


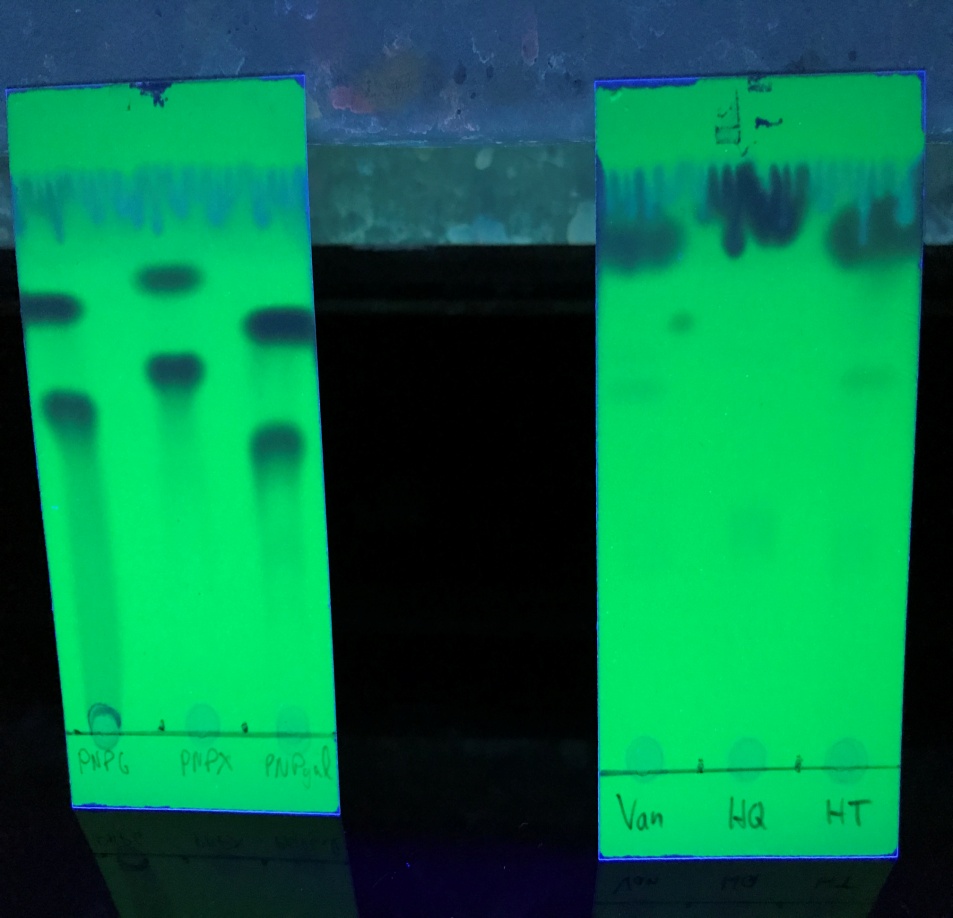


**C**


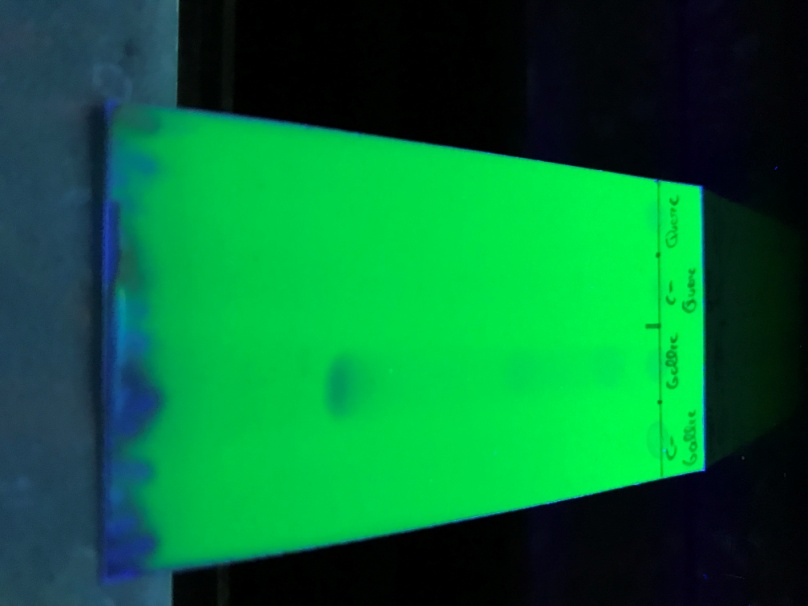


**A**

**Figure S2.** Thin layer chromatography of the different reaction mixtures in transglycosylation reactions using BGL-1-E521G. A) Gallic acid reaction. B) Nitrophenyl sugars reaction. C) Vanillin, hydroquinone and hydroxytyrosol reactions. D) EGCG reaction. The new products detected are signaled with arrows.

BG1 QEVYITTTGYTARPQCTEPPATPTFRFQSFSYASLNDTIRYAISVPSPTTTHTYGPAYTD 60

Termite ------------------------------------------------------------ 0

Clostridium ------------------------------------------------------------ 0

Trichoderma ------------------------------------------------------------ 0

BG1 AVAKLSTKLTTTTWGSWVPSQTVISATDTADKYGQAAWSSQWLHASLANYTNIGLYTTTV 120

Termite ------------------------------------------------------------ 0

Clostridium ------------------------------------------------------------ 0

Trichoderma ------------------------------------------------------------ 0

BG1 NPTPLPSSELVLPPRDYFGPTDCYNFPEGFTFGVAGSAAQIEGAIGLEGRAPSILEKLLP 180

Termite --------------MDVASSDTVYTFPDEFKLGAATASYQIEGAWDENGKGPNIWDTLTH 46

Clostridium --------------------MEKLRFPKDFIFGTATAAYQIEGAYKEDEKGESIWDRFSH 40

Trichoderma -----------------MHHHHHHMLPKDFQWGFATAAYQIEGAVDQDGRGPSIWDTFCA 43

:*. * * * :: ***** : :. .* : :

BG1 DT------EPQDYVTNENYYLYKQDIQRLASXGVKYYSFSISWGRILPFTVPGSPINEQG 234

Termite EHPDYVVDGATGDIADDSYHLYKEDVKILKELGAQVYRFSISWARVLPEGH-DNIVNQDG 105

Clostridium -IPGNVAKMHNGDIACDHYHRYKEDVQLLKSLGIKSYRFSIAWPRIFPKGF--GEINQKG 97

Trichoderma -QPGKIADGSSGVTACDSYNRTAEDIALLKSLGAKSYRFSISWSRIIPEGGRGDAVNQAG 102

. : : * :*: * . * : * ***:* *::* . :*: *

BG1 LKHYNDLIDYVLEVGMVPIVTMLHFDTPLYFINASAGYVVPDIGYQNGGYWNK-EFVDSF 293

Termite IDYYNNLINELLANGIEPMVTMYHWDLPQALQDL-------------GGWPNL-VLAKYS 151

Clostridium IQFYRDLIDELIKNDIEPAITIYHWDLPQKLQDI-------------GGWANP-QVADYY 143

Trichoderma IDHYVKFVDDLLDAGITPFITLFHWDLPEGLHQR------------YGGLLNRTEFPLDF 150

:..* .::: :: .: * :*: *:* * : : ** * .

BG1 VNYGKILFTHFADRVPFWVTINEPLLYAFN----------------------FTGLDNVV 331

Termite ENYARVLFKNFGDRVKLWLTFNEPLTFMDGYASEIGMAPSINTPGIGD----YLAAHTVI 207

Clostridium VDYANLLFREFGDRVKTWITHNEPWVAS-----YLGYALGVHAPGIKDMKMALLAAHNIL 198

Trichoderma ENYARVMFRAL-PKVRNWITFNEPLCSA-----IPGYGSGTFAPGRQSTSEPWTVGHNIL 204

:*..::* : :* *:* *** ..::

BG1 HAHAELYHFYHDT---LNGTGKVGLKLNDNFGVPKHPENQTEIDAANRFNDMQLGVFAYP 388

Termite HAHARIYHLYDQEFR-AEQGGKVGISLNINWCEPATN-SAEDRASCENYQQFNLGLYAHP 265

Clostridium LSHFKAVKAYRE----LEQDGQIGITLNLSTCYSNS-ADEEDIAAAHRSDGWNNRWFLDA 253

Trichoderma VAHGRAVKAYRDDFKPASGDGQIGIVLNGDFTYPWDAADPADKEAAERRLEFFTAWFADP 264

:* . : * : . *::*: ** . . : :... :

BG1 ICL-GQQYPKSILDTLPG-----------AKPLSKKELEYISHTTDFIGIDAYTATVISV 436

Termite IFTEEGDYPAVLKDRVSRNSADEGYTDSRLPQFTAEEVEYIRGTHDFLGINFYTALLGKS 325

Clostridium AL--KGTYPEDMIKIFSD--------TNIMPELPKELFTEVFETSDFLGINYYTRQVVKN 303

Trichoderma IY--LGDYPASMRKQLGD--------R--LPTFTPEERALVHGSNDFYGMNHYTSNYIRH 312

** : . . : : : : ** *:: **

BG1 PAEGIEYCSKQ---NMTTNSLYPYCVTQETVNSYGWDIGYRSQSYVYITPTYLRAFLSYI 493

Termite GVEGYEPSRYR-DSGVILTQ---DA----------WP--ISASSWLKVVPWGFRKELNWI 370

Clostridium NSEAFIGA-----ESV-------AMD---------NP--KTEMGWE-IYPQGLYDLLTRI 339

Trichoderma RSSPASADDTVGNVDVLFTNKQGNCI---------GP--ETQSPWLRPCAAGFRDFLVWI 361

. .: : : * *

BG1 WNTYKT-PLVLSEFGFPVYAESTRDLVDQLYDSPRSQYYLSFMSELLKSIWEDGVDVIGA 552

Termite KNEYNNPPVFITENGFSDY--------GGLNDTGRVHYYTEHLKEMLKAIHEDGVNVIGY 422

Clostridium HRDYGNIDLYITENGAAFNDMVN--RDGKVEDENRLDYLYTHFAAALSIEAGVPLKGY 396

Trichoderma SKRYGYPPIYVTENGTSIKGESDLPKEKILEDDFRVKYYNEYIRAMVTAVELDGVNVKGY 421

. * : ::* * : * * .* .: :.: ** : *

BG1 IAWSFMDNWEFG-DYAQQFGMQVVNRTTQE-RWFKK-SFFDIVDFVGARNGLGYAAASFL 609

Termite TAWSLMDNFEWLRGYSEKFGIYAVDFEDPARPRIPKESAKVLAEIMNTRKIPERFRDLEH 482

Clostridium YIWSFMDNFEWAEGYEKRFGIVHVNYKTQE-RTIKK-SAYWYKELIERSNKLEHHHHHH- 453

Trichoderma FAWSLMDNFEWADGYVTRFGVTYVDYENGQ-KRFPKKSAKSLKPLFD------------- 467

**:***:*: .* :**: *: : * * :.

BG1 EQKLISEEDLNSAVD------ 630

Termite HHHHH---------------- 487

Clostridium --------------------- 453

Trichoderma --------------------- 467

**Figure S3.** Alignment of BGL-1 amino acid sequence with the BGLs sequences of bacterium *Clostridium cellulovorans*, fungus *Trichoderma reesei* and termite *Neotermes koshunensis*. The catalytic amino acids are highlighted in red. The alignment was performed using Clustal omega, with default settings.


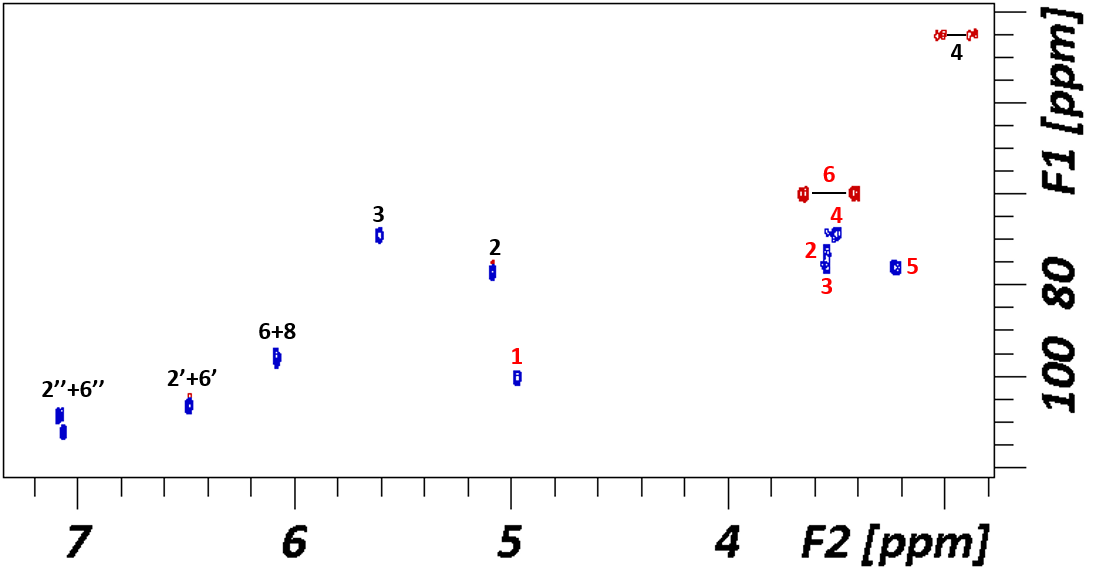


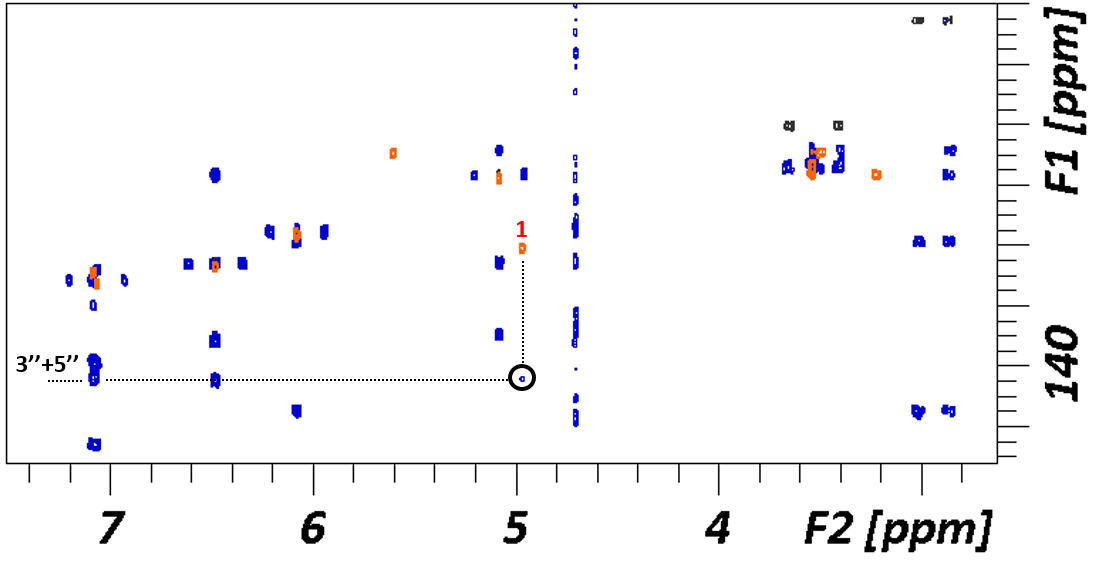


**Figure S4.** NMR Spectra of the EGCG glycoside. **Top:** Labelled ^1^H-^13^C HSQC of EGCG-glucose. **Bottom:** Superimposition of ^1^H-^13^C HSQC (black/orange) and HMBC (blue) spectra. The correlation of the anomeric position is highlighted.


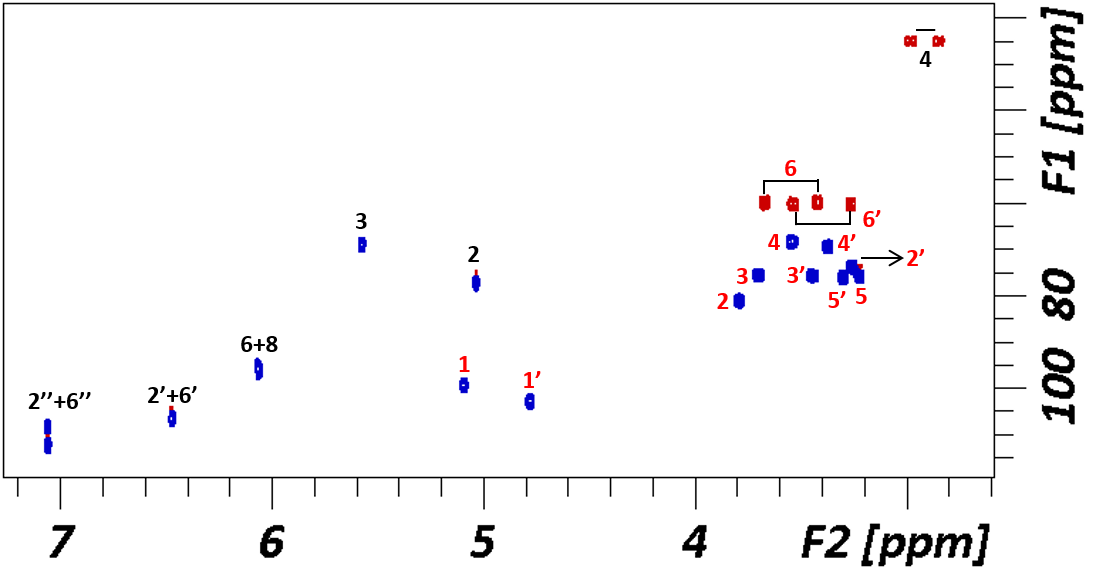


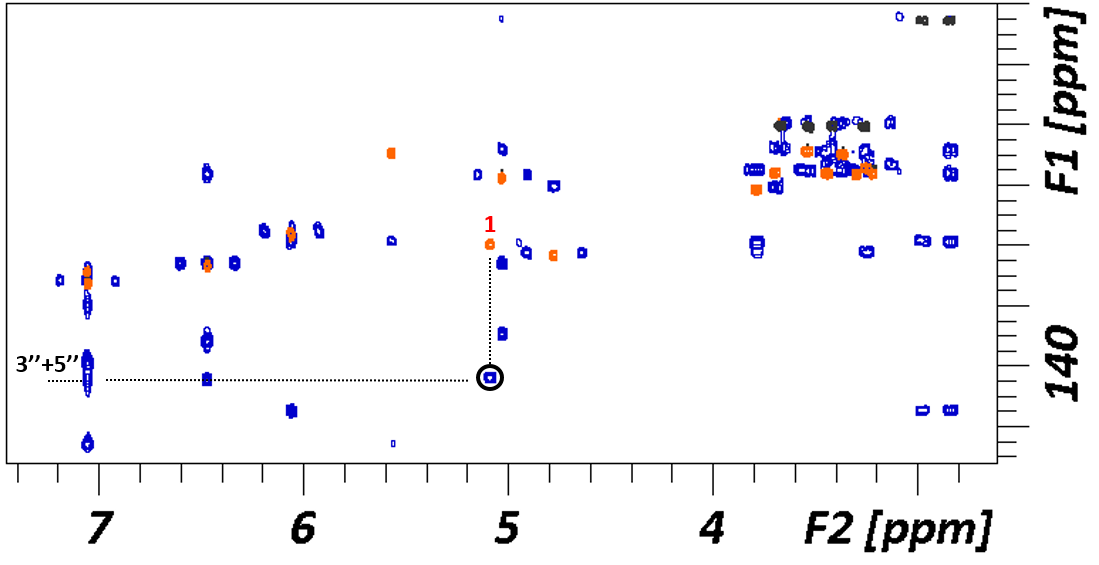


**Figure S5.** NMR Spectra of the EGCG diglycoside. **Top:** Labelled ^1^H-^13^C HSQC of EGCG-sophorose. **Bottom:** Superimposition of ^1^H-^13^C HSQC (black/orange) and HMBC (blue) spectra. The correlation of the anomeric position is highlighted.

**
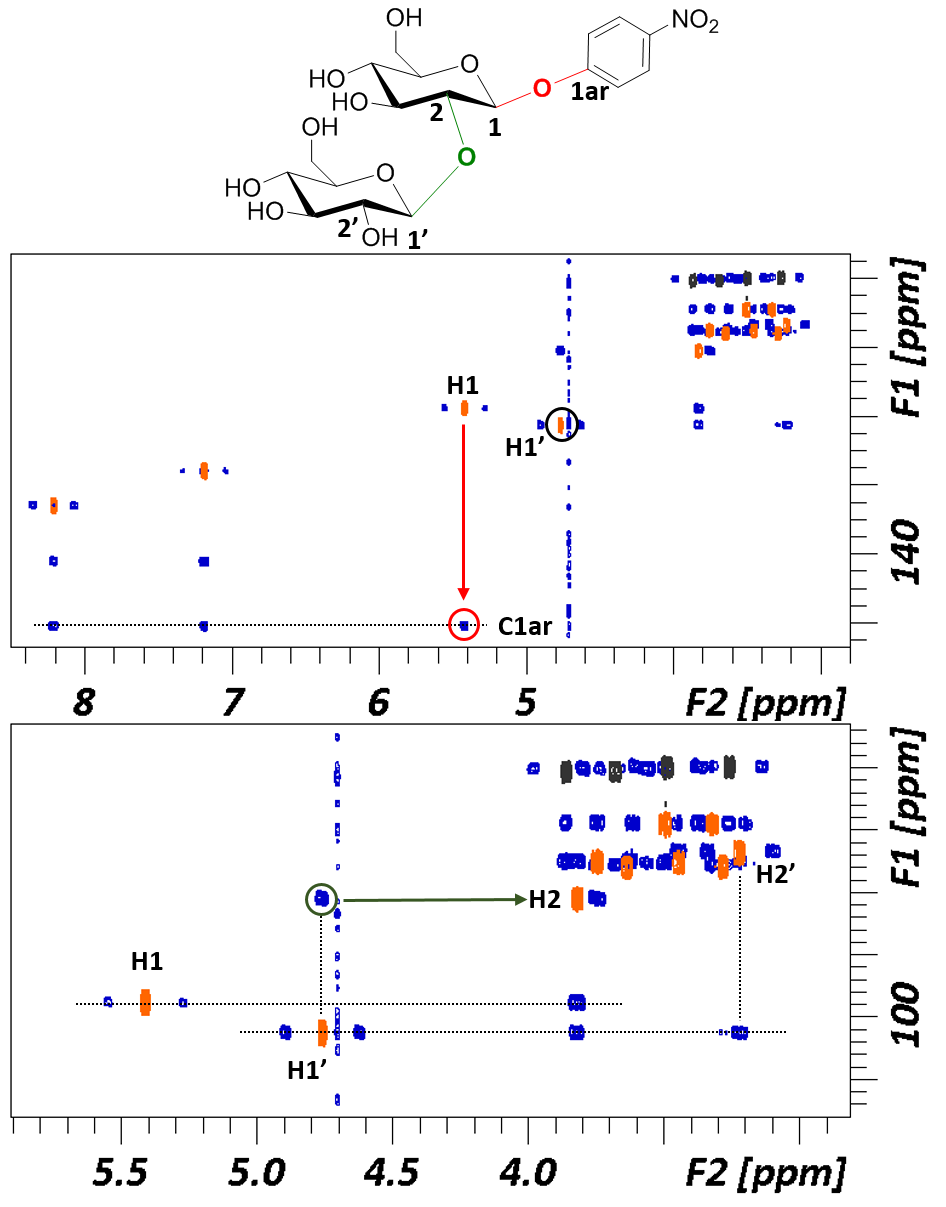
**

**A**

**
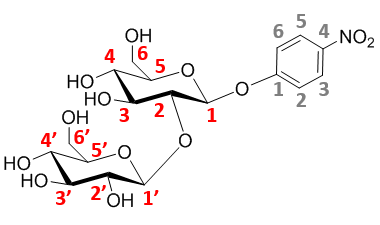

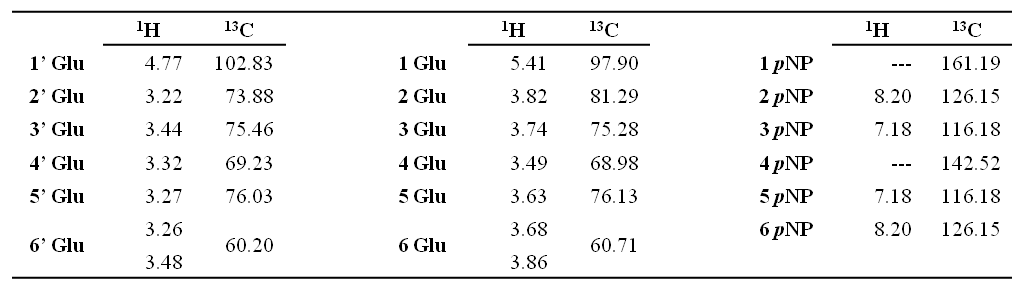
**

**B**

**Figure S6.** **A)** Top: structure of the Glucose-Glucose-*p*NP derivative. Superimposition of ^1^H-^13^C HSQC (orange/black) and HMBC (blue). Bottom: Expansion of the carbohydrate region. Arrows represent the key cross peaks for the characterization of the molecule. The lower panel shows the correlation of the anomeric H1’ proton of the transferred glucose with the carbon at position 2 in the acceptor glucose for the disaccharide linkage (green), and the upper panel displays the correlation of the anomeric position of the acceptor glucose and the quaternary carbon of the *p*NP ring residue (red). **B)** Structure, deduced from NMR analysis, of the *p*NPG-glucoside produced by transglycosylation of *p*NPG with the synthase BGL-1-E521G. Every C atom in the molecules is numbered to clarify the identification of the signals. Chemical shifts (ppm) are indicated in the table.

**
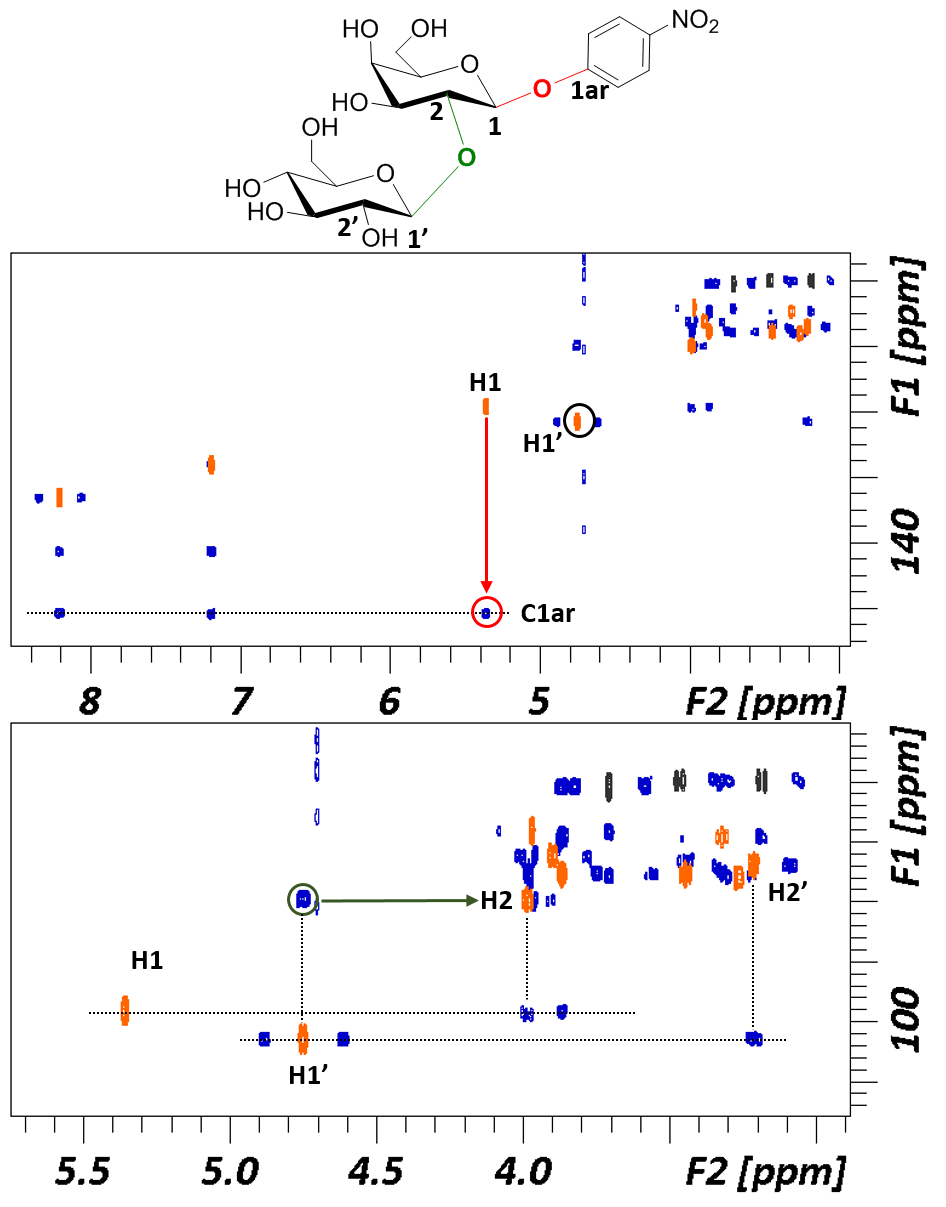
**

**A**

**
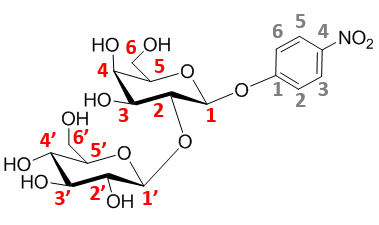

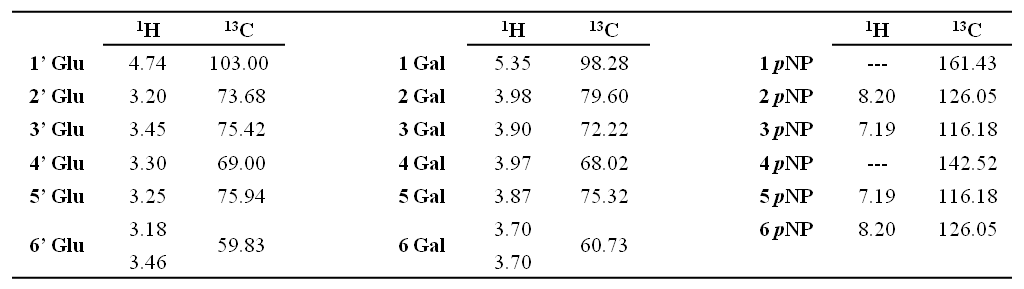
**

**Figure S7.** **A)** Top: structure of the Glucose-Galactose-*p*NP derivative. Superimposition of ^1^H-^13^C HSQC (orange/black) and HMBC (blue). Bottom: Expansion of the carbohydrate region. Arrows represent the key cross peaks for the characterization of the molecule. The lower panel shows the correlation of the anomeric H1’ proton of the transferred glucose with the carbon at position 2 in the acceptor galactose for the disaccharide linkage (green), and the upper panel displays the correlation of the anomeric position of the acceptor galactose and the quaternary carbon of the *p*NP ring residue (red). **B)** Structure, deduced from NMR analysis, of the *p*NPGal-glucose product obtained by transglycosylation of *p*NPGal with the synthase BGL-1-E521G. Every C atom in the molecule is numbered to clarify the identification of the signals. Chemical shifts (ppm) are indicated in the table.

**B**


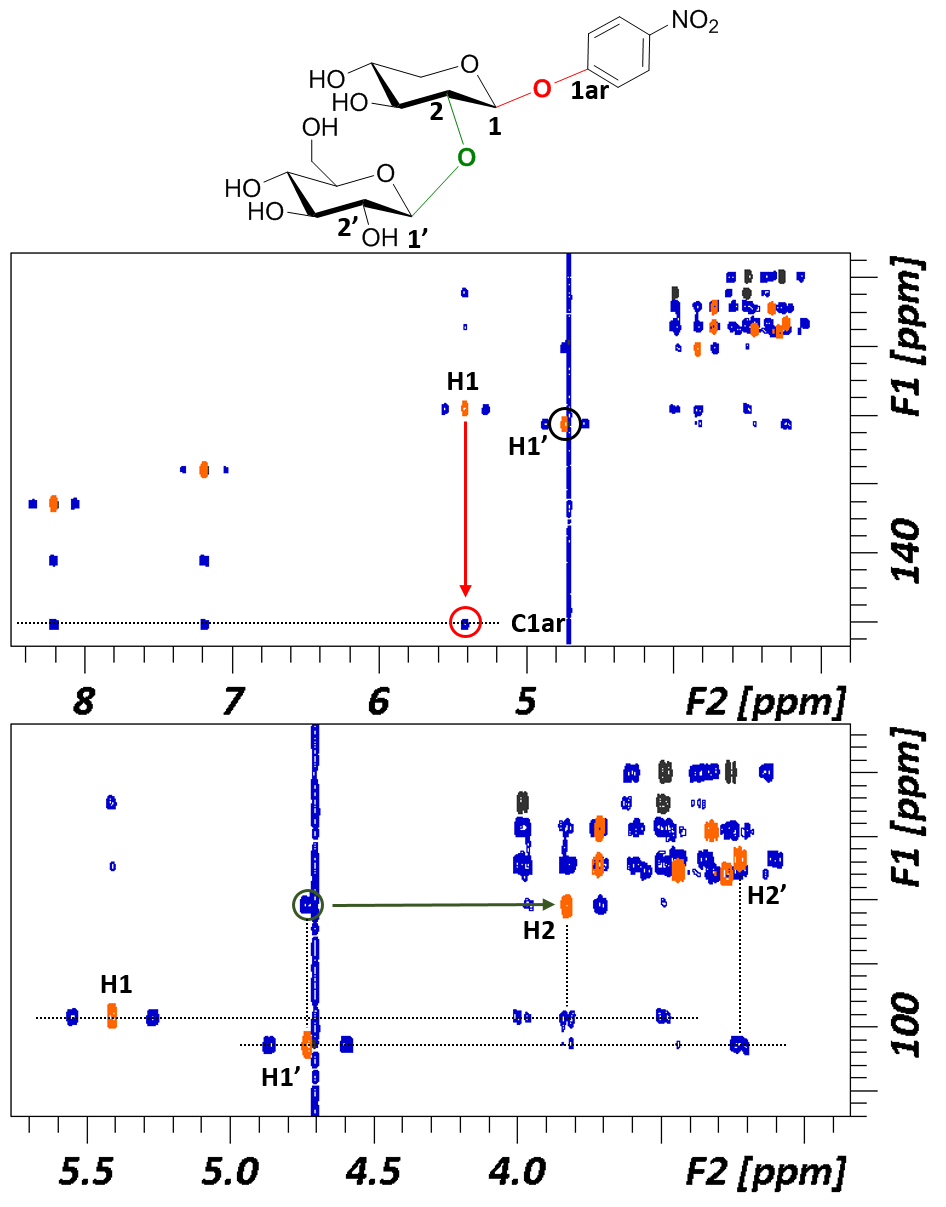


**A**

**
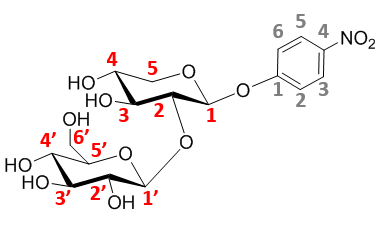

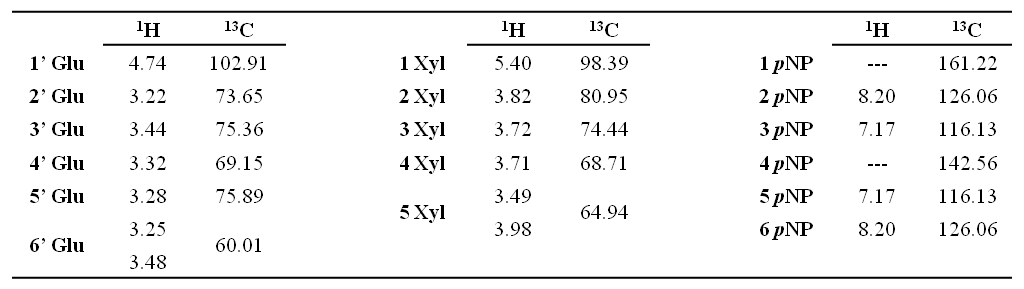
**

**B**

**Figure S8.** **A)** Top: structure of the Glucose-Xylose-*p*NP derivative. Superimposition of ^1^H-^13^C HSQC (orange/black) and HMBC (blue). Bottom: Expansion of the carbohydrate region. Arrows represent the key cross peaks for the characterization of the molecule. The lower panel shows the correlation of the anomeric H1’ proton of the transferred glucose with the carbon at position 2 in the acceptor xylose for the disaccharide linkage (green), and the upper panel displays the correlation of the anomeric position of the acceptor xylose and the quaternary carbon of the *p*NP ring residue (red). **B)** Structure, deduced from NMR analysis, of the *p*NPX-glucose product obtained by transglycosylation of *p*NPX with the synthase BGL-1-E521G. Every C atom in the molecule is numbered to clarify the identification of the signals. Chemical shifts (ppm) are indicated in the table.

**Figure S9.** Kinetic calculations of inhibition constant of BGL-1 by glucose.

|  |  |  |  |  |
| --- | --- | --- | --- | --- |
| **Glucose concentration (mM)** | **Inhibited *K_m_*** | ***K_i_*** | **Average (mM)** | **Standard deviation (mM)** |
| **0** | 3.72* |  |  |  |
| **1000** | 4.68 | 3875 | 3780.4 | 136.9 |
| **1250** | 4.93 | 3842.9 |  |  |
| **1500** | 5.26 | 3623.3 |  |  |

* Normal *K_m_* calculated in absence of glucose

*K_m_* of purified BGL-1 was calculated in the presence of 1, 1.25, and 1.5 M glucose using *p*NPG (10 μM to 5 mM). *K_i_* was determined using the formula:

$Ki=Normal Km*\frac{Glucose concentration}{Inhibited Km-Normal Km}$
